# Drug supply situation in Rwanda during COVID-19: issues, efforts and challenges

**DOI:** 10.1186/s40545-021-00301-2

**Published:** 2021-01-20

**Authors:** Theogene Uwizeyimana, Hashim Talib Hashim, Jean Damascene Kabakambira, Jean Claude Mujyarugamba, Jackson Dushime, Blaise Ntacyabukura, Remy Ndayizeye, Yusuff Adebayo Adebisi, Don Eliseo Lucero-Prisno

**Affiliations:** 1 Department of Public Health, Mount Kenya University Rwanda, Kigali, Rwanda; 2 College of Medicine, University of Baghdad, Baghdad, Iraq; 3 Department of Internal Medicine, University Teaching Hospital of Kigali, Kigali, Rwanda; 4 Department of Pharmaceutics and Pharmacy Practice, University of Nairobi, Nairobi, Kenya; 5 Global Health Focus Africa, Kigali, Rwanda; 6 Global Public Health Department, Karolinska Institutet, Solna, Sweden; 7 Institute for Public Health Innovation, Richmond, VA USA; 8 Faculty of Pharmacy, University of Ibadan, Ibadan, Nigeria; 9 Department of Global Health and Development, London School of Hygiene and Tropical Medicine, London, UK

**Keywords:** Drug supply system, COVID-19, Drug import, Rwanda

## Abstract

COVID-19 is a threat to health systems around the world and Rwanda is not an exception. The impact of the pandemic is far-reaching and access to health commodities is not spared. Proper drug supply is critical for a robust healthcare system. It determines the extent at which the population are likely to have access to essential medicines and treatments. In Rwanda, the pharmaceutical sector heavily relies on imports. With the emergence of COVID-19 pandemic, the drug supply system was interrupted leaving many stores from small local pharmacies to the big medical stores running out of stock. The reasons were limited importation of goods from abroad, and the panic buying practice among the customers and some institutions when responding to the pandemic. Drug and medicines accessibility, availability and affordability should be the core of any drug management policy. It is with no doubt that, Rwanda has made a tremendous work to mitigate the effect of COVID-19 on the country’s drug supply; however, efforts are still needed to invest in local pharmaceutical production as a way to minimize import expenses in the country. Good policy on drug importation, production and distribution should be enforced to avoid any drug shortage that may be encountered in the Rwandan drug market.

## Background

COVID-19 is a global public health threat affecting many countries around the world and Rwanda is not an exception. The impact of the pandemic is far-reaching and access to health commodities is not spared. Proper drug supply is critical for a robust healthcare system. It determines the extent at which the population are likely to have access to essential medicines and treatments. In Rwanda, the pharmaceutical sector heavily relies on imports [1]. In 2019, Rwanda spent around $ 97.6 million on drugs import alone, and it is expected to increase by $102.5 million in 2024 [1]. About 20% of these import expenses are expected to be reduced by the establishment of the first pharmaceutical factory in the country [2]. Public and private sectors preserve the drug supply chain in Rwanda. In public sector, the drug supply chain system allows public health institutions to obtain health commodities directly from central medical store and/or district pharmacies [3]. The Medical Production and Procurement Division (MPPD) under Rwanda Biomedical Centre (RBC), performs the procurement, storage, and distribution of health commodities to public health institutions and it is regarded as central medical store. MPPD is the main supplier of all pharmaceutical commodities ranging from essential medicines to laboratory reagents. However, district pharmacies serve as the distribution point of pharmaceutical products to district hospitals and health centres. Public sector can also get an authorization from the Ministry of Health or/and district pharmacies that allow them to buy pharmaceutical commodities from private wholesalers in case they are not in their stores. In private sector, many wholesale pharmaceutical companies import drugs directly from international manufacturers, and local drug compounding pharmacies. Community pharmacies and private health clinics or hospitals get medical commodities from these private wholesalers.

With the emergence of COVID-19 pandemic, the drug supply system was interrupted leaving many stores from small local pharmacies to the big medical stores running out of stock. The reasons were limited importation of goods from abroad (mainly china and India), and the panic buying practice among the customers and some institutions when responding to the pandemic, and avoiding falling in possible health commodities shortage. The government of Rwanda restricted the movement of people crossing the borders, but allowed the passage of goods and cargo as a way to keep the continuous supply of goods including health commodities. However, since the demand is too high and no current local drug manufacturers to compensate the available gap, the drug shortage continues to be a major issue in some settings. Drug and medicines accessibility, availability and affordability should be the core of any drug management policy [4]; however, it is unwarranted to consider that in a country with limited local manufacturing. This paper aims at providing the details on current situation of drug supply chain in Rwanda, and the efforts and shortfalls faced in times of COVID-19 pandemic.

## Current efforts

Despite Rwanda being affected by the current COVID-19 pandemic, different global institutions and bodies have recognized the efforts made to contain the spread of the virus. Maintaining the continuous supply of health commodities to COVID-19 patients as well as general population is the challenging issue that requires regular monitoring. However, Rwanda realized that it could only deal with such scenario through a properly coordinated and multi-sectoral approach [5]. Many factors influence the current efforts to maintain the country’s drug supply amid the pandemic.

## Political commitment

At the beginning of the COVID-19 pandemic, a multi-ministerial committee was established, and led by the prime minister. The team is not only dealing with the implementation of COVID-19 response and containment plans, but also ensures the continuous availability of essential health commodities to the COVID-19 patients, frontline workers as well as general population. Rwanda welcomed the support of several partners, and made advisory team of scientists to help the COVID-19 Joint Task Force (JTF) [6]. The panic practice of buying too many drugs and other health commodities that was adopted by Rwandese due to the pandemic was controlled. This was done by establishing the regulations that require pharmacists to serve customers with limited quantities per person and with proper judgements. The government endorsed the local manufacturing of COVID-19 response equipment ranging from hand sanitizers, to other personal protective equipment (PPE). The security of the country was maintained and all pharmacies, hospitals and many other essential businesses were greenlighted to continue the services but using 50% of the employees to avoid overcrowding. This allowed the patients on long-term treatments and chronic diseases to access drugs when needed. Rwanda is blessed with good governance and strong cooperation between its citizens and local leaders, and this has hugely contributed to good responses made to contain the virus and ensure medicine security is prioritized.

## Economic stability

Prior to the COVID-19 pandemic, Rwanda was among the Africa’s fastest growing economies. Economic growth exceeded 10% in 2019, and the strong growth was expected in 2020. This was highly contributed by the large public investments in the implementation of the national strategy of transformation [7]. Many pharmaceutical companies are currently investing in the country, with ApexBiotech being the first company licensed by Rwanda Food and Drug Authority (RFDA) to start its operations, and it is currently in the final establishment phase [2]. In a bid to avoid the redirection of fiscal resources to the COVID-19 emergency response, the World Bank Group provided Rwanda with a credit of $ 14.25 million to fund the Rwanda COVID-19 Emergency Response Project [7]. Rwanda has a clear economic plan that attracts foreign investors, and pharmaceutical sector is facing a considerable growth in this case.

## Price control

Medicines were generally affordable in Rwanda prior to the pandemic. The prices of essential medicines and drugs in public sectors are lower compared to the international procurement prices. In private sectors, the prices are double, but still it is 30% below the international reference prices [8]. During the pandemic, the country faced an increase in drug and health commodities demand with limited importations, and this resulted in some prices of the pharmaceutical products being raised above the normal range. However, the Rwandan Ministry of Trade and Industry warned, inspected and fined any company that raised the prices of essential commodities including pharmaceuticals amid the pandemic [9]. The list of pharmaceuticals on high demand was publicly posted with their prices and qualities, and pharmacies were requested to comply with it.

## Privatization of pharmaceutical sector

Rwanda is currently privatizing the pharmaceutical sector. This is because the current regulations restrict MPPD from contacting and importing pharmaceuticals directly from international manufacturers. In this case, MPPD uses intermediate suppliers that import drugs and other pharmaceutical products from manufacturers and sell them with increased prices that make health commodities quite expensive for public sectors. Therefore, Rwanda Medical Supply (RMS) is in its introductory phase to replace MPPD [10]. RMS will serve as the central medical store that has its full authorization to negotiate prices and import directly from international manufacturers. District pharmacies will continue to serve as a distribution points and warehouses of RMS, and public health institutions will obtain all pharmaceutical products and medical equipment directly from there. District pharmacies will no longer be commercial entities, and this will reduce the pharmaceutical costs as well as public healthcare cost in general. The adaptation of the privatization policy will reduce the lead-time of pharmaceuticals as well as mitigate the country’s drug supply shortage.

## Challenges

Rwanda like many other African countries relies on China and India for their medicine security [11]. This has been a trending issue for years and with the COVID-19 pandemic, the scenario worsened. The pharmaceutical industry is among the hardest hit by the COVID-19 pandemic, due to the increase in demand with limited access to low materials. In Rwanda, drug supply chain is mainly challenged by lack of enough local drug manufacturers and high dependence on international manufacturers. Although the country has made it easy to start and operate businesses for both local and foreigners, there is a huge investment gap in pharmaceutical sector. Lack of capacity to manufacture good formulations because of skills gap on product development and formulation expertise, is not only the Rwandan challenge, but also for the whole East African Community (EAC) [12]. Even though, most of East African countries borders were closed during the pandemic, the trade of essential businesses like food and pharmaceuticals were waived, allowing Rwanda to continue the consumption of drugs manufactured in other EAC member states. However, due to the fact that Kenyan pharmaceutical sector was also challenged by limited access to low materials and the fight to satisfy its local market [13], Rwanda pharmaceutical supply shortage persisted.

Indian-owned pharmaceutical companies in Rwanda are the ones that dominate the market. They sell drugs to local wholesalers at reasonable prices and local wholesalers add some amount of money as a profit since they are commercial entities. This brings uncertainty in the drug market, and with COVID-19, many products became high-priced. During the time of COVID-19, all the big pharmaceutical companies, whether Indian or locally owned, were challenged by the export restrictions imposed by India and many other supplying countries [14]. This led to shortage of drugs and other health commodities in the Rwandan market. The prices of different drugs increased regardless of whether they are selling new stocks, or the stocks made before the pandemic. Some local pharmaceutical companies have shifted their tension from making their owned local products, to manufacturing hand sanitizers, facemasks and many other PPEs that are currently needed in the fight against COVID-19. This has led to complete shortage of some products that are usually needed by the population. Since other non-essential businesses were closed, Rwanda is also facing an upsurge in new pharmacy businesses, which increases the pharmaceuticals demand and competition in the market. [15] The delay of ApexBiotech pharmaceutical company to start its operation that was expected in April 2020 has also contributed to the existing medicines and drug shortage, as it was to reduce 20% of pharmaceutical import.

## Conclusion

Rwanda, like many other African countries, needs to strengthen the pharmaceutical sector. It is with no doubt that, Rwanda has made a tremendous work to mitigate the effect of COVID-19 on the country’s drug supply; however, efforts are still needed to invest in local pharmaceutical production as a way to minimize import expenses in the country. Good policy on drug importation, production and distribution should be enforced to avoid any drug shortage that may be encountered in the Rwandan drug market. We also recommend that Rwanda should invest in herbal medicine research towards ensuring medicine security in the country.

## Data Availability

Not applicable.

## Abbreviations

MPPD: Medical Production and Procurement Division
RBC: Rwanda Biomedical Centre
JTF: Joint Task Force
PPE: Personal protective equipment
RFDA: Rwanda Food and Drug Authority
RMS: Rwanda Medical Supply
EAC: East African Community
